# Impact of electrode–tissue proximity on formation of pulsed field ablation lesions: Using multi-electrode impedance measurements

**DOI:** 10.1016/j.hroo.2025.05.026

**Published:** 2025-06-02

**Authors:** Marijn H.A. Groen, Jeffrey M. Fish, Peter Loh, René van Es

**Affiliations:** 1 Department of Cardiology, University Medical Center Utrecht, Utrecht, The Netherlands; 2 Abbott Laboratories, St. Paul, Minnesota

**Keywords:** Pulsed field ablation, Cardiac ablation, Contact measurements, Efficacy, Pigs

## Abstract

**Background:**

For pulsed field ablation (PFA), the effect of electrode–tissue contact or proximity remains unclear.

**Objective:**

In this porcine study, the effect of electrode–tissue proximity was studied after 3 weeks of follow-up, using a custom device in which the electrode–tissue distance could be varied for every electrode.

**Methods:**

An impedance-based method was used to determine electrode–tissue proximity or contact. During a bench experiment, the relation between contact value per electrode and electrode–tissue distance was studied. In addition, in 10 pigs epicardial ablation using a custom 6-polar linear ablation device was performed using bipolar, biphasic deliveries on randomized locations on the heart. After 3 weeks, macroscopic analysis was used to compare lesion width for left vs right ventricle. Based on histology, the lesion area and depth were related to electrode–tissue contact.

**Results:**

Analysis of 96 bench measurements showed a decrease in tissue-proximity index with increasing distance. Lesions on the right ventricle were wider compared to the lesions on the left ventricle, 9.0 ± 1.9 mm vs 6.5 ± 1.0 mm (*P* < .001), respectively. A total of 162 lesions showed that a higher tissue-proximity index was associated with deeper and larger lesions; lesion depth was 1.3 ± 0.5 mm vs 1.5 ± 0.5 mm (*P* = .016) and lesion area 6.4 ± 2.7 mm^2^ vs 8.3 ± 2.9 mm^2^ (*P* < .001) for low vs high indices respectively.

**Conclusion:**

This study shows that higher electrode–tissue proximity may result in deeper PFA lesions, and is, therefore, an important parameter in improving PFA lesions. Therefore, confirmation of tissue contact using impedance-based measurements may improve PFA ablation efficacy in a clinical setting.

## Graphical abstract

**Figure:**
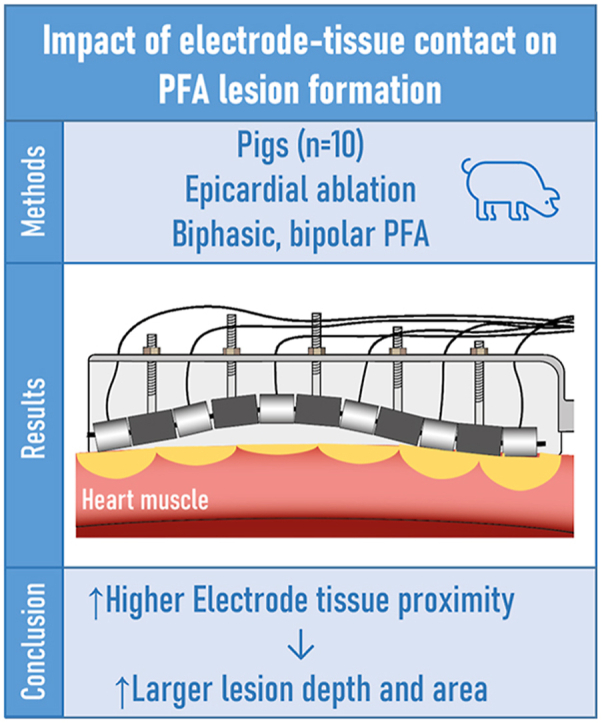

**Key Findings**

▪ In a porcine study after a 3-week follow-up period, pulsed field ablation (PFA) lesions on the right ventricle were wider compared to lesions on the left ventricle (9.0 ± 1.9 mm vs 6.5 ± 1.0 mm).
▪ Upon histology, a higher tissue-proximity index used during PFA ablation was associated with deeper and larger lesions (lesion depth was 1.3 ± 0.5 mm vs 1.5 ± 0.5 mm [*P* = .016], and lesion area 6.4 ± 2.7 mm^2^ vs 8.3 ± 2.9 mm^2^).
▪ Confirmation of tissue contact using impedance-based measurements may improve PFA ablation efficacy in a clinical setting.

## Introduction

Pulsed field ablation (PFA) is an emerging ablation method to perform cardiac ablation through irreversible electroporation.^1^, ^2^, ^3^, ^4^ With PFA, an electric current is applied between 2 or more electrodes or between a catheter and an indifferent skin patch. Above a certain threshold, current density will lead to cell death. The efficacy of PFA ablation is dependent on the local current density rather than the transfer of heat. Therefore, while thermal ablation efficacy is partially dependent on applied pressure by the electrode to the target tissue,^5^ it is thought that PFA ablation is more influenced by the distance between the electrode and the tissue rather than the actual contact force.

Moreover, recent studies focused on the influence of contact force on PFA lesion formation, but contact-force measurements cannot be implemented in the often-used multi-electrode catheter with complex shapes.^6^, ^7^, ^8^ Other studies found that tissue proximity is a valuable indicator of lesion efficacy during PFA.^9^, ^10^, ^11^ However, these studies were either acute, based on vegetable models, or including multiple applications in complex endocardial ablation settings. Therefore, more research is required to establish well-founded conclusions regarding the effect of tissue proximity/contact.

The multi-electrode impedance system (MEIS) is an electrical impedance-based method to determine electrode–tissue proximity and contact.^12^^,^^13^ Since this measurement does not involve contact-force sensors, this technique is also suitable for multi-electrode (circular) catheters. As it is an impedance-based measurement, no actual contact force is measured. In this porcine study, the goal was to study the effect of electrode–tissue proximity after 3 weeks of follow-up, using a custom investigational device in which the electrode–tissue distance can be varied for every electrode separately. Electrode–tissue proximity or contact was determined using the MEIS impedance system.

## Methods

### Bench—MEIS vs electrode distance

**Figure 1:**
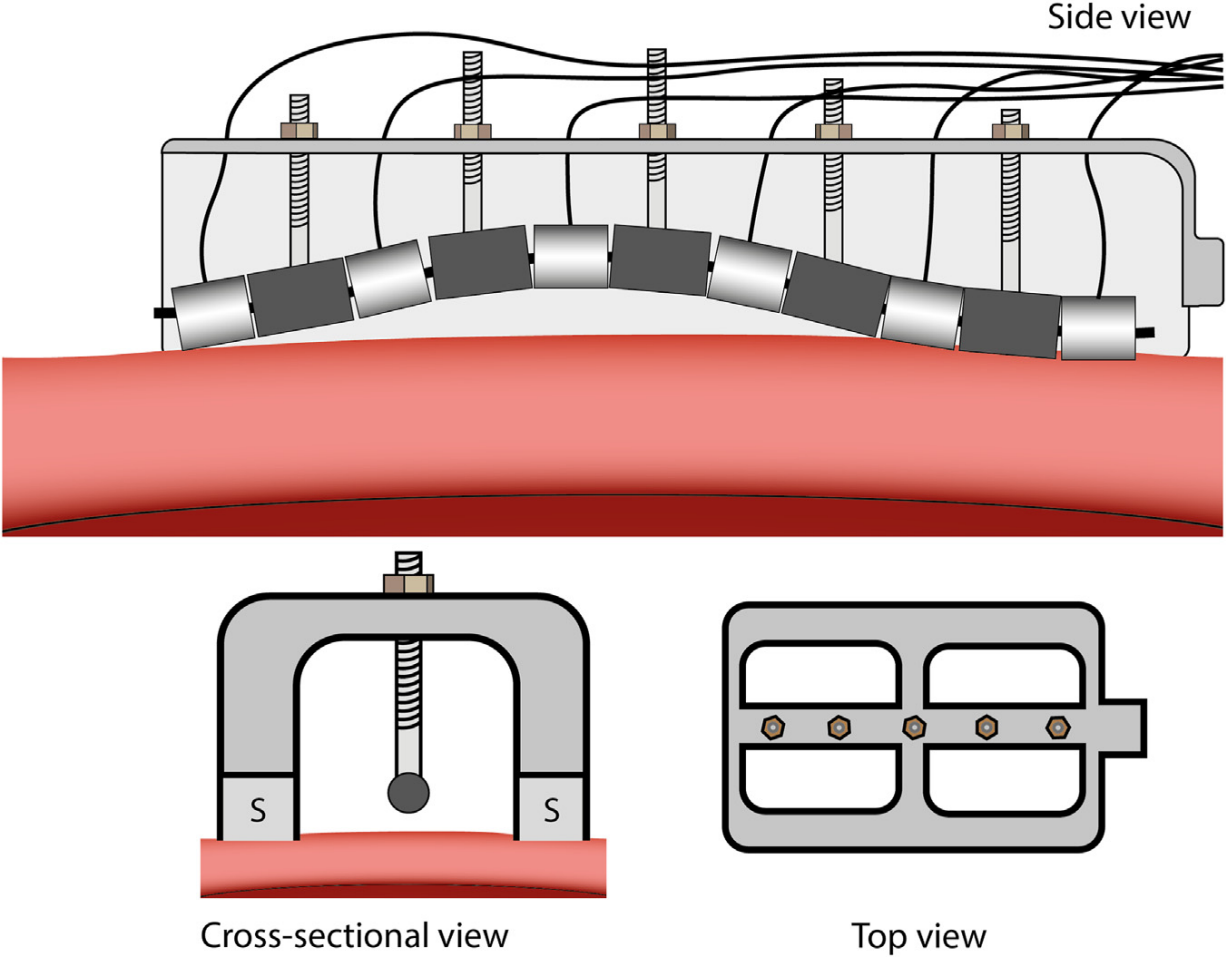
Catheter device set up including side-, cross-sectional- and top view. A 6-polar (2.5 mm electrodes, 4 mm spacing) linear catheter was attached to a 3D-printed device including 2 parallel suction cups (S) for vacuum. Between every electrode, a screw was fixated and secured with a nut on the top of the device. Electrode–tissue distance can be adapted by adjusting the nuts. 3D = 3-dimensional.

A bench experiment was performed to study the relation between MEIS value per electrode and electrode–tissue distance. A translucent tank was filled with a mixture of 500 mL 0.9% saline to 700 mL sterile water to reach a target conductivity of 7 mS/cm (similar to blood). A custom 6-polar linear ablation device with 2.5 mm electrodes with 4 mm spacing (Figure 1) was positioned parallel to a slice of freshly excised porcine left ventricle. With MEIS, a small drive current is applied between 2 neighboring electrodes (electrode pair 1-2, 3-4, and 5-6), while measuring the voltage between the target electrode and an indifferent skin patch.^12^^,^^13^ Baseline MEIS values were measured with the electrodes in contact with the tissue. After that, the ablation device was moved away from the tissue in steps of 0.5 mm until a stable MEIS value was reached. This procedure was repeated 4 times using different tissue slices and electrode distance configurations.

### Animal study

All experiments were approved by the Animal Experimentation Committee of the University Medical Center Utrecht and are following the Guide for Care and Use of Laboratory Animals.^14^

For this study, 10 female Topigs Norsvin pigs (60–70 kg) were used. The animals were given 1200 mg Amiodarone per day starting 7 days before the procedure and 800 mg per day during the follow-up period to prevent heart rhythm disorders as a result of the epicardial procedure, as it is known (and in our experience) that pigs are very susceptible to arrhythmias due to, for example, device manipulation. Pre-procedure, a buprenorphine plaster (20 mg), midazolam (0.4 mg/kg), atropine (0.05 mg/kg) and ketamine (10 mg/kg) were administered. At the start of the procedure, the animals were sedated, anesthetized, and intubated using thiopental sodium (4 mg/kg), midazolam (0.5 mg/kg/h), sufentanil (2.5 ug/kg/h) and cis-atracurium (0.7 mg/kg/h). A midline sternotomy was performed, and the heart was exposed for further experiments.

### Ablation setup

**Figure 2:**
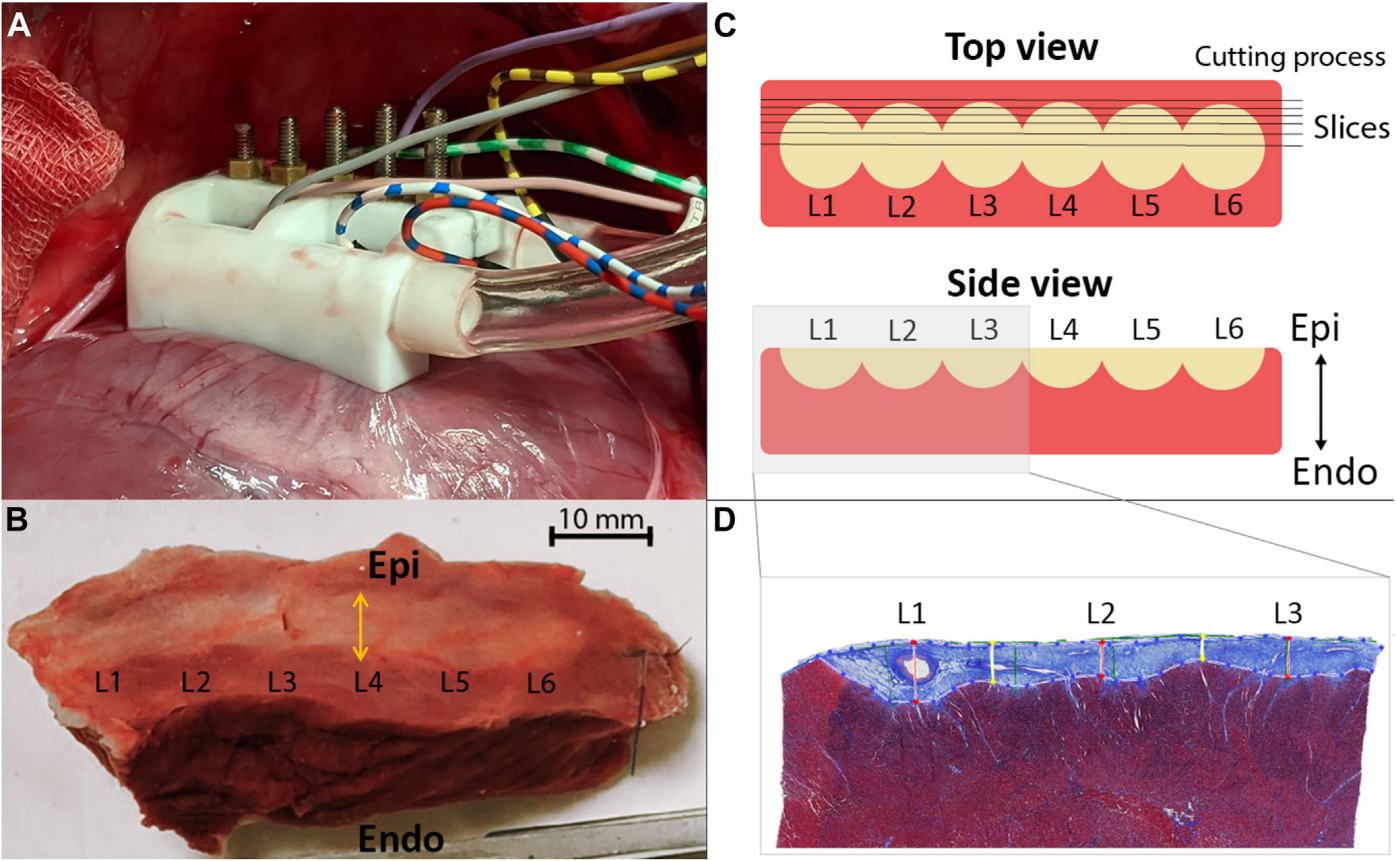
**A:** Image of the positioning of the catheter device on the Epi surface. **B:** Top view of a macroscopic lesion of an application on the left ventricle after 3 weeks of follow-up. L1–L6 = lesion 1–6, *yellow arrow* = example of a width measurement **C:** Schematic overview of the cutting process, multiple slices spaced 500 μm apart are cut along the catheter. **D:** Example of histological analysis (Masson’s trichrome) in which 3 lesions are displayed. *Blue tissue* = lesions, *Red line* = lesion depth, *yellow line* = lesion edge. Endo = endocardial; Epi = epicardial.

The custom 6-polar linear ablation device was positioned at the epicardium using a vacuum with an underpressure of 50–60 cm H_2_O applied to the 2 suction cups parallel to the catheter (Figures 1 and 2A). Two epicardial lesions were created on the left ventricle and 1 on the right ventricle. For every ablation position, the electrode–tissue distances per electrode were randomly adjusted between 0 and 3 mm (Figure 1). Ablation was performed using bipolar (electrode 1-3-5 vs 2-4-6), and bi-phasic deliveries using 25 bursts with 40 pulses per burst (1000 pulses in total) at 1400V. MEIS was used as a surrogate measurement for electrode–tissue contact. During MEIS measurements and during ablation, the thorax cavity was filled with the same mixture of 500 mL 0.9% saline to 700 mL sterile water as in the bench experiment.

### Data collection

During the procedure, epicardial electrograms and surface electrocardiograms were recorded using Workmate Claris (Abbott, MN). Raw MEIS signals were recorded for 5 seconds pre- and post-ablation, MEIS data was synchronized with cardiac signals and electrocardiogram. Over the 5 seconds pre- and post-ablation, the mean MEIS value was calculated per electrode. Per ablation, a reference MEIS measurement using the same catheter was performed in a bowl with the same saline/water mixture to compensate for any deviations between electrodes or connections. For analysis, the measured MEIS value minus the reference value per electrode was used, which will be referred to as the tissue-proximity index. After the procedure, the animals were closed up.

### Follow-up

A follow-up period of 3 weeks was used to allow for lesion maturation. After that, the heart was removed, and the lesions were excised and fixated. Lesions were photographed for macroscopic analysis (Figure 2B). After fixation, the lesions were cut in slices spaced 500 μm apart parallel to the ablation line starting at the edge of the lesion (based on macroscopic identification of the lesion) and stained using hematoxylin and eosin stain and Elastica van Gieson or Masson’s trichrome (Figure 2C and 2D).

Using macroscopic images, the maximum lesion width (perpendicular to the catheter) per application was measured by an experienced researcher. Based on histology, lesion depth, and lesion area along the catheter at maximum depth were measured per electrode and used for analysis. Lesions that were located on epicardial fat were excluded from the analysis.

### Statistical analysis

All continuous variables are expressed as mean value ± standard deviation. Groups were compared using an independent samples *t* test. A linear mixed model with random intercept per animal and corrected for delivered current was used to study the relation between MEIS values and lesion depth and area using R and SPSS statistics. Statistical significance was accepted at *P <* .05.

## Results

### Bench—MEIS vs distance

**Figure 3:**
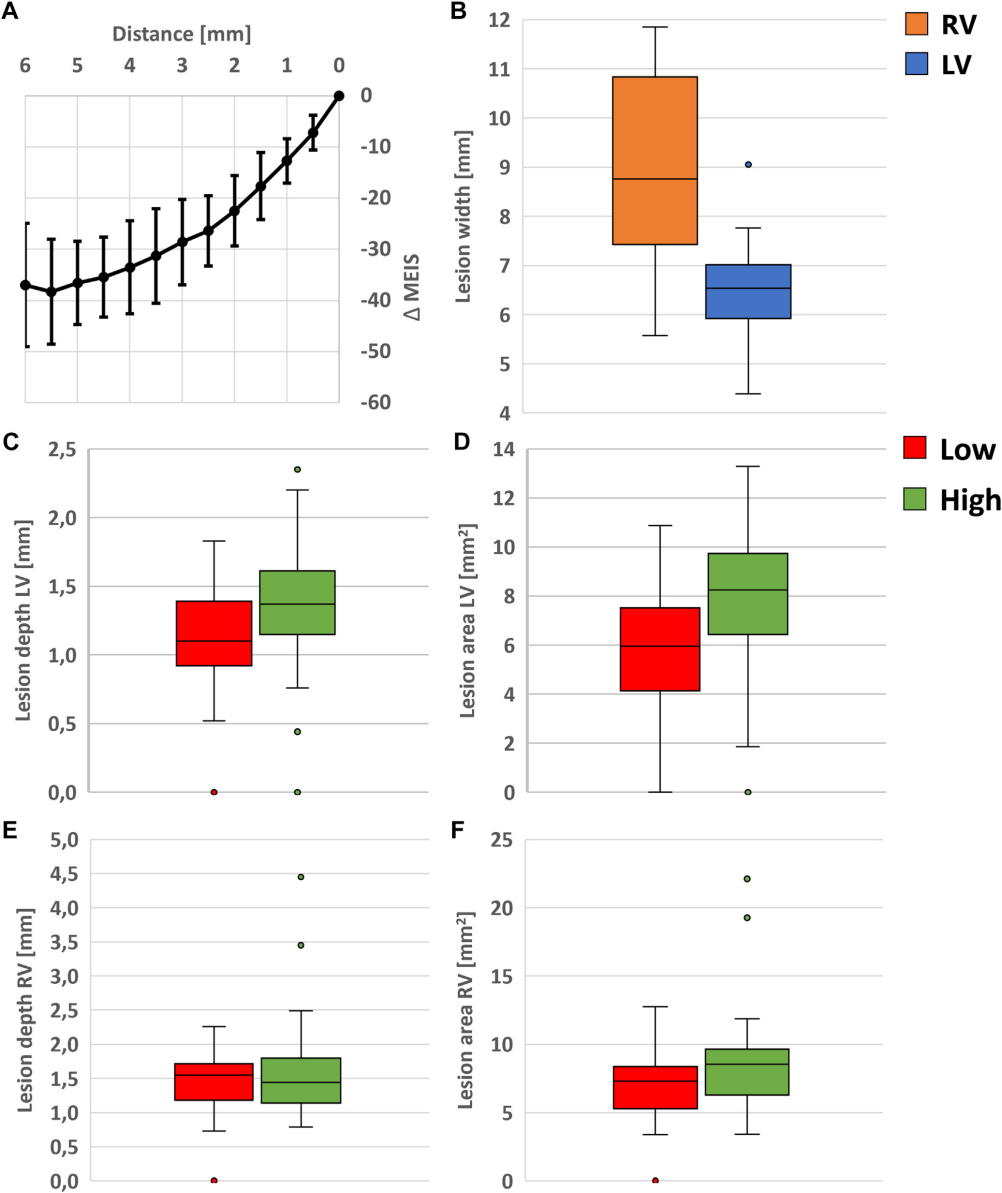
**A:** Electrode–tissue distance (x-axis) vs Δ MEIS (relative value to full contact MEIS-value). **B:** Lesion width LV vs RV based on macroscopic analysis. **C and D:** Lesion depth and area for the LV for low vs high MEIS value, respectively. **E and F:** Lesion depth and area for the RV for low vs high MEIS value, respectively. LV = left ventricle; MEIS = multi-electrode impedance system; RV = right ventricle.

A total of 96 measurements were analyzed. A decrease in MEIS values with increasing distance relative to the baseline (linear relation of −10.5 Ohm [Ω]/mm in the first 3 mm) with the electrode in full contact was seen, with a flattening of the curve starting around 3 mm distance (Figure 3A).

### Animal study

All animals survived the procedure and follow-up period without complications. All measurements were performed by an investigator who was blinded for the tissue-proximity index for the specific lesions.

### Macroscopic analysis

Directly after ablation, the lesions were visible as white discoloration. After follow-up, most of the lesions were clearly visible as white discoloration and tissue shrinkage at the separate electrodes (Figure 2A).

On a total of 25 locations (16 left ventricle [LV] and 9 right ventricle [RV]), maximum lesion width per application was determined. Lesions on the right ventricle were wider compared to the lesions on the left ventricle, 9.0 ± 1.9 mm vs 6.5 ± 1.0 mm (*P* < .001), respectively (Figure 3B). There was no significant difference in the delivered total current for LV and RV applications (25 ± 3A and 26 ± 2, respectively, *P* = .299). There was no significant difference in the tissue-proximity index between applications on the LV and RV (21.4 ± 16 Ω and 16.9 ± 14 Ω, respectively, *P* = .087).

### MEIS threshold

Tissue-proximity indexes were scored as low or high with a threshold of 13.5 Ω. The overall MEIS value post-ablation was significantly lower compared to the MEIS value pre-ablation with a ratio of pre/post = 1.10 ± 0.1, *P <* .001.

### Lesion depth and area

None of the applications resulted in transmural lesions. In the cases where electrodes were positioned on epicardial fat, lesions could still be observed histologically. However, there was no clear demarcation between the lesion and pre-existing fatty and fibrotic tissue. These lesions were excluded from further analysis. No damage was observed to the vessels within the lesions, as was previously described by Du Pré and colleagues.^15^

A total of 161 lesions were used for the analysis, of which 57 were on the RV and 104 were on the LV. Overall, the mean lesion depth was 1.4 mm, and the mean lesion area was 7.7 mm^2^. For low vs high tissue-proximity index, for the LV a significant difference in lesion depth (1.1 ± 0.4 mm vs 1.4 ± 0.4 mm, *P* = .002) and lesion area (6.0 ± 2.5 mm^2^ vs 8.2 ± 2.4 mm^2^, *P* < .001) was found (Figure 3C and 3D). For the RV, no significant difference in lesion depth (1.4 ± 0.5 mm vs 1.6 ± 0.7 mm [*P* = .08]) and lesion area (7.0 ± 2.7 mm^2^ vs 8.7 ± 3.6 mm^2^ [*P* = .46]) was found (Figure 3E and 3F).

Overall, a significant linear relation was found between tissue-proximity value and lesion depth and area (R^2^ = 0.233, *P* = .002 and R^2^ = 0.193, *P* < .001, respectively).

## Discussion

In this study, we used a long-term follow-up porcine model in combination with a custom linear catheter device to study the impact of electrode–tissue proximity on lesion formation using PFA ablation. It was shown that higher electrode–tissue proximity led to deeper lesions on the LV, suggesting that this is an important parameter for improving PFA ablation efficacy in a clinical setting.

During the bench experiment, a decrease in the MEIS values was seen with an increasing electrode–tissue distance. However, in comparison with a previous study using a tip-electrode catheter the curve was not as steep for distances close to tissue contact.^12^ This might be explained by the fact that in this experiment, a linear multi-electrode catheter was used. Therefore, the neighboring electrode can influence the MEIS measurement for the target electrode. Nevertheless, a clear decrease in MEIS value was seen with an increasing electrode–tissue distance.

Based on macroscopic analysis, it was found that lesions placed on the RV were significantly wider compared to lesions on the LV. Moreover, no significant differences were found between lesion depth and area on the RV for low vs high tissue-proximity index. It was hypothesized that since the thickness of the RV wall is considerably lower compared to the LV wall, this will lead to a more focused electric field within the tissue. Thereby, the lesion width is smaller for a thicker tissue wall. Moreover, as the RV is relatively thin and less stiff, the vacuum system as used in this catheter device leads to more tissue between the 2 suction cups. This might increase the tissue that is exposed to the electric field. Moreover, at the RV there is less distance toward the blood pool underneath, which has a higher conductivity compared to the tissue. Therefore, we hypothesize that the results on the LV are more reliable using this specific catheter setup. Further studies are required to study the differences between PFA ablation on the LV and RV, to develop an optimal pulse protocol for both locations.

We found that the MEIS value directly post-ablation is lower compared to the MEIS value pre-ablation. This was as expected since a lower impedance is a direct effect of the PFA ablation as also suggested by other studies.^16^ Changes in local impedance might be used as a marker for lesion formation during PFA ablation.

In this study, the relatively small lesions could explain that there is only a small difference between lesions with a lower vs higher tissue-proximity index (on average 1.3 vs 1.5 mm, respectively). This is a result of the chosen pulse parameters as energy and number of applications. As in this study, the goal was to compare the different lesion sizes per electrode it was not desired to create transmural lesions. Therefore, we chose to perform 1 application per ablation location. Despite the small lesion sizes, a significantly lower lesion depth and the area was found for lesions with a lower vs higher tissue-proximity index on the LV, suggesting that electrode–tissue proximity is an important factor for lesion efficacy. In this study, a mean lesion depth of 1.4 mm was found, which in a clinical setting is not sufficient to create transmural lesions. In a clinical setting, pulse parameters should be adjusted in such a way that overall lesion depth is increased.

Although the contact force between the electrode and tissue does not directly impact lesion formation, applying increased pressure can push the electrode into the tissue, forming a depression in the tissue in which the electrode sits. This, in turn, increases the electrode and the tissue contact area, most likely influencing the formation of lesions. In the present study, we did not assess this effect.

### Clinical implications

PFA ablation is an upcoming technology to perform cardiac ablation. Although the primary results from clinical trials are promising, there is not yet a consensus about the importance of electrode–tissue proximity. Moreover, the golden standard method for the commonly used radiofrequency ablation techniques to measure electrode–tissue contact, namely contact-force measurements, is not suitable for multi-electrode catheters. With the MEIS system, local impedances can be electrically measured per electrode for multiple electrodes at the same time. Using the results of this study, it is shown that increasing the electrode–tissue proximity as measured by the MEIS system can increase ablation efficacy. Clinical trials are required to further confirm these results.

## Limitations

Relatively small lesions were created during these experiments, therefore there were only small differences. There might have been a larger difference between lesions with a low vs high tissue-proximity index if the overall lesion depth was larger. In addition, in a clinical setting variations of the distance between electrode and tissue might be larger compared to the set up in this study. This might lead to larger differences in lesion depth.

In this study, a specific bipolar, biphasic PFA protocol was used, which might be different from pulse protocols used for other systems. Because little is known about the specific pulse protocols used by the different PFA systems, it is not known whether the results of electrode–tissue proximity as measured with the MEIS system will have the same impact for all different pulse protocols. Nevertheless, electrode–tissue proximity is directly related to the current density within the target tissue and is therefore thought to influence ablation efficacy regardless of the pulse protocol.

In these experiments, epicardial ablation was performed using an investigational ablation device. We used a water/saline solution with a conductivity comparable to blood, to mimic endocardial conditions. However, the endocardial tissue surface is not as smooth as the epicardial surface, which may affect the results. Therefore, it should be considered that a translation is required toward endocardial ablation. Moreover, the use of the reference measurements as performed before each ablation is not the preferred method during clinical use. In the case of endocardial ablation, reference measurements can be performed in 2 positions, namely with and without tissue contact with the endocardial wall.

## Conclusion

This long-term porcine study shows that higher MEIS values and thus higher electrode–tissue proximity will lead to deeper PFA lesions in a bipolar configuration, and may therefore be an important parameter to improve PFA ablation efficacy in a clinical setting.

## Disclosures

Peter Loh is a consultant for Abbott. René van Es filed patents regarding the ablation technology. Jeff Fish is an employee at Abbott and filed patents regarding the ablation technology. Marijn Groen has no conflicts of interest to disclose.
